# Fear of Death, Emotional Intelligence and Resilience Among Healthcare Staff During COVID-19: A Correlative Study

**DOI:** 10.1155/jonm/7872841

**Published:** 2025-05-06

**Authors:** Sara Martínez-Jabares, Ana I. López-Alonso, Natalia Calvo-Ayuso, Guillermo Charneco-Salguero, Marta Quiñones-Pérez, María Cristina Martínez-Fernández

**Affiliations:** ^1^Department of Nursing, Faculty of Medicine, San Pablo-CEU University, CEU Universities, Madrid, Spain; ^2^HeQoL Research Group, Nursing and Physiotherapy Department, Faculty of Health Sciences, University of León, León, Spain; ^3^SALBIS Research Group, Faculty of Health Sciences, Department of Nursing and Physiotherapy, Campus of Ponferrada, University of León, León, Spain; ^4^Emergency Department, University Health Care Complex of León (CAULE), León, Spain; ^5^HeQoL Research Group, Faculty of Health Sciences, Department of Nursing and Physiotherapy, Campus of Ponferrada, University of León, León, Spain

**Keywords:** COVID-19, emotional intelligence, fear of death, health professionals, resilience

## Abstract

The suffering, pain and fear of death experienced by patients during the pandemic have evoked a wide range of feelings and emotions in healthcare professionals. Managing these emotions is influenced by factors such as emotional intelligence and resilience. Recognising and addressing these emotions can enhance the quality of care and help prevent mental health issues. This study analyses the relationship among fear of death, emotional intelligence and resilience, as along with the sociodemographic variables of healthcare professionals during the COVID-19 pandemic. A descriptive, comparative and correlational study was conducted. Participants included professionals from the Neurosurgery, Pneumology, Emergency Service and Floating Staff units in a tertiary-level hospital in Spain. Data were collected through an anonymous and voluntary online survey, which included sociodemographic data, Collett–Lester fear of death, emotional intelligence and resilience scale. Participation was anonymous and voluntary. A total of 121 professionals participated, predominantly women (85.12%), with a mean age of 41.89 years (SD ± 9.6). Nurses compromised 64% of the sample. Fear of others' death (37.2%) and the dying process of others (33.1%) increased. Emotional intelligence levels were generally adequate across all work areas, with women scoring higher in attention and clarity. However, significant differences in emotional regulation were observed between groups. Resilience scores were high across all participants (> 78.77). Positive correlations were found among age, fear of the dying process of others (*p*=0.003) and resilience (*p*=0.002). An inverse correlation was observed between fear of others' death and resilience (*p*=0.018) and emotional regulation (*p*=0.020). Linear regression analysis identified attention to emotions, acceptance and bioethics training as predictors of fear of death.

## 1. Introduction

The global pandemic of COVID-19 has had a significant impact on Spain, leading to considerable chaos and uncertainty due to the high rate of infection and mortality [1]. The crisis management strategy was primarily focussed on preventing the further spread of the virus within the community, reducing the number of hospitalisations and deaths. This approach placed additional stress on healthcare staff [2, 3]. This context has been found to trigger a few psychological problems experienced by health professionals, including anxiety, fear, depression and stress [4]. A study by Ruiz-Frutos and Gómez-Salgado [5] identified several factors that contributed to the mental instability of health professionals. These include individual and collective elements, such as fear of contagion, shortages of protective equipment, work overload and social pressure [5]. This has resulted in challenges in decision-making in complex situations, leading to professional distancing and burnout, particularly among frontline professionals [6–8]. These factors have had a negative impact on the quality of patient care and the well-being of health professionals [9]. The recent research has identified the necessity of addressing the mental health of health professionals in order to mitigate the effects of the ongoing health crisis, with management of both individual and collective factors being a key.

The inevitability of death, a fundamental aspect of life, evokes a range of emotions and attitudes in individuals as they face their own mortality based on their psychological state, personal experiences and the meaning they attach to life and death [10]. This situation was further exacerbated during pandemic times [11]. Healthcare professionals, who accompany individuals during the process of dying, also experience irrational fears about this event, which can lead them to avoid or distance themselves from their professional duties [7, 8].

Studies have been carried out on professionals working in palliative care units where they are regularly involved in end-of-life situations. Self-care and awareness are known to positively predict professionals' competence to cope with dying, and this, together with awareness, positively predicts compassion satisfaction and negatively predicts compassion fatigue and burnout [12]. The ongoing pandemic has emphasised the need to prioritise the mental health of healthcare professionals, who regularly encounter the fear of death in their daily work [6, 13].

Emotional intelligence (EI) plays a crucial role in healthcare settings, as it enables professionals to recognise and regulate their emotions. It also acts as a protective factor, fostering more resilient responses, such as positive adaptation to stressful environments [14, 15]. EI has shown to be a key factor for nurses in effective decision-making and positive behaviour in challenging situations. In contrast, those with low EI have been found to experience heightened stress and emotional conflict, which can hinder their ability to adapt to change or social interactions [16]. During the COVID-19 pandemic, healthcare workers have reported challenges in managing their emotions, emphasising the need to develop EI [17–19]. Furthermore, EI is essential in managing experiences related to death, both for healthcare professionals and for patients, providing the necessary skills to navigate the emotional complexities associated with it. This enhances not only personal well-being but also the quality of patient care. In this context, training in EI is essential for health professionals, as it enables them to manage their emotional responses to the death of patients and to improve their communication and leadership skills. This, in turn, enables them to better support patients and their families during end-of-life care [20]. Numerous studies have demonstrated the pivotal role of EI in enhancing not only patient care and work dynamics [14] but also emotional well-being and conflict resolution [21]. Structured EI programmes have been shown to improve stress management and job satisfaction, as well as promote emotional awareness and regulation [22, 23]. In addition, simulation-based training has been shown to be effective in identifying emotional triggers and improving conflict resolution skills, helping to combat what is known as ‘emotional deafness' in healthcare settings [24]. Conversely, a lack of EI can generate negative emotions such as anxiety and stress, which affect both the quality of patient care and professional satisfaction [4, 25, 26].

Resilience is defined as a set of personal competencies, such as self-esteem, optimism, coping strategies and social support, which enable individuals to cope with extreme situations, adapt flexibly to adversity, recover from challenges and maintain good mental health [27, 28]. However, the precise definition of resilience remains complex, complicating efforts to achieve a comprehensive understanding [29]. Nevertheless, resilience is influenced by personal, environmental and social factors, which can either facilitate or impede its development [30]. Research indicates that, at the individual level, positive thinking, affect and coping, realism and behavioural control are factors that promote resilience. At the family level, family support is the key, while at the unit level, positive leadership is essential. Finally, at the community level, belonging is a crucial factor [31]. In the context of temporal considerations, two distinct forms of resilience can be identified. Firstly, acceptance resilience, which is predicated on a resource conservation strategy, is particularly well-suited to minor, short-term adversities. Secondly, strategic resilience, which is contingent on a resource strategy, is arguably more appropriate for protracted challenges. Additionally, significant individual differences are evident, with time and context playing critical roles [32]. In the field of mental health, resilience was extensively studied during the COVID-19 pandemic [13, 33]. The literature points to the close relationship between EI and resilience, both of which are associated with reduced stress and increased job satisfaction [13, 34]. Facing multiple adversities at both physical [35] and environmental [36] levels generated anxiety in health professionals [37]. However, these challenges have also exposed individuals' resilience, which enables the development of new resources and strengths. Viejo et al. [38] and Mendoza Bernal [14] have examined the impact of health, psychological and social factors on resilience, while Castagnola Sánchez et al. [37] have identified characteristics of resilient people. This suggests that experiencing the pandemic may enhance resilience for future adversities. Armangué and Crespo [39] recognise that social factors, such as cultural and political relations, influence resilience, opening new research avenues during the pandemic.

## 2. Aim

The aim of this study was to analyse the relationship among fear of death, EI and resilience among healthcare professionals during the course of COVID-19 pandemic.

## 3. Methods

A cross-sectional correlational study was conducted using online surveys. The study population comprised healthcare professionals from the neurosurgery, pulmonology and emergency units of a public hospital in Spain. The sample size was estimated at 127 responses from a total population of 188 professionals, with a confidence level of 95%. Ultimately, 121 complete and voluntary responses were analysed. The participants included doctors, nurses and nursing assistants who were actively working during the data collection period (January–March 2022). Professionals from other categories and hospital units were excluded.

### 3.1. Variables and Measurement Instruments

An ad hoc questionnaire was developed to collect sociodemographic data (age, gender, academic training and work experience). Additionally, the Fear of Death Scale (FDS), EI and the resilience scale (ES) were included.

The FDS, or Brief Fear of Death Scale (BFODS), was developed by Collett and Lester in 1969. This multidimensional instrument consists of 28 items divided into four subscales: Fear of One's Own Death, Fear of the Death of Others, Fear of One's Own Dying Process and Fear of the Dying Process of Others. Each item uses a five-point Likert-type response format, ranging from ‘*I do not worry at all*' (1 point) to *‘I worry a lot*' (5 points) [40]. High mean scores on subscales indicate high fear levels, while low mean scores suggests low fear levels. Scores are categorised as *low fear* (< 2), *moderate fear* (≥ 2–4) and *high fear* (> 4 to 5) [41]. This scale, validated across various cultural contexts, shows acceptable psychometric properties [42].

The Emotional Intelligence Scale (TMMS-24), adapted into Spanish by Fernandez-Berrocal et al. [43], is a condensed version of the original TMMS-48 by Salovey et al. [44]. It consists of 24 items measuring three areas: attention to feelings, understanding of feelings and emotional repair capacity. Responses are rated on a five-point Likert scale, ranging from 1 (*strongly disagree*) to 5 (*strongly agree*) [43]. Each dimension is scored independently using pre-established cutoff points by sex (based on consistent and statistically significant differences observed in validation studies of the scale, showing that emotional socialisation and gender stereotypes persist to this day). The TMMS-24 has proven to be an instrument that offers adequate levels of reliability and validity to be used in EI assessment/training processes [45].

The ES-14, originally developed in English by Wagnild and Young in 1993, and revised in 2005, was validated in Spanish by Teruel and Bello [46]. The scale assesses two factors: Personal Competence (self-confidence, independence, decisiveness, resourcefulness and perseverance) and Acceptance of Self and Life (adaptability, balance, flexibility and a stable outlook). Responses are rated on a Likert scale from 1 (*strongly disagree*) to 7 (*strongly agree*). Resilience levels are classified as very high (98–82), high (81–64), normal (63–49), low (48–31) and very low (30–14) [33].

### 3.2. Procedure

Data were collected in an online format due to the circumstances of the ongoing pandemic. Participants received a link to the survey via Lime Survey. The researcher informed unit supervisors, who then distributed the survey to the relevant staff via WhatsApp. Further reminders were sent out in February and March to encourage participation.

### 3.3. Statistical Analysis

Descriptive analysis was conducted. Quantitative variables were described using the mean and standard deviation, while qualitative variables were described using frequencies, percentages and standard errors. Statistical tests such as the *t*-Student test and analysis of variance (ANOVA) were used to contrast differences between quantitative variables. Pearson's coefficient was used to assess associations between quantitative variables. A forward logistic regression model was implemented to predict fear of death based on the variables of interest. Tests with a *p* value < 0.05 were considered significant. Data analysis and processing were performed using SPSS Statistics 27, and tables and graphs were generated using Excel 2023.

### 3.4. Ethical Considerations

Medical and nursing heads were informed and invited to participate in the study, disseminating the survey among their teams. Participants completed the survey voluntarily and anonymously after being informed of its purpose. Confidentiality was ensured through an accompanying consent form. They had the option to view results. Approval was obtained from the University Hospital's Clinical Research Ethics Committee (Internal Registry No. 21181), adhering to ethical principles outlined in the Declaration of Helsinki, European Data Protection Regulation and Law 3/2018 on Data Protection and Digital Rights and Guarantees.

## 4. Results

### 4.1. Sociodemographic Descriptive Data

The participation rate was 60.6%. The mean age was 42 ± 9.63 years, and 85.12% were women. On average, participants had worked for 14.95 ± 8.64 years. Further descriptive information on the sample is presented in Table 1.

All scales used in the study showed high reliability, as indicated by Cronbach's alpha coefficients. The RS showed high reliability, as evidenced by a Cronbach's alpha coefficient of 0.817. The Emotional Intelligence Scale had a coefficient of 0.905 and the Collett–Lester FDS showed a Cronbach's alpha coefficient of 0.941.

The percentages were calculated using the cut-off points of each scale in order to divide the participants into meaningful categories according to the interpretation standards of the different scales. The questionnaires with this information are presented in Tables 2, 3 and 4.

### 4.2. Descriptive Scales

#### 4.2.1. Means and Standard Deviations of the Level of Fear/Anxiety Towards Death, EI and Resilience Categorised by Job Position

Table 5 presents the means and standard deviations obtained on the scales. The mean resilience score (82.57 ± 8.37) falls within the very high resilience range (98–82), with floating staff professionals obtaining a lower score (78.76 ± 10.81), still within the high resilience range. On the EI scale, professionals in all work areas demonstrated adequate attention levels, with women generally achieving higher scores, particularly those in the pneumology unit and floating staff (30.50 ± 6.04 and 30.69 ± 7.18). Similar trends were observed in the understanding area, with values indicating adequate understanding and women achieving higher scores, especially in the pneumology and floating staff units (30.25 ± 5.92 and 30.15 ± 5.17). In terms of emotional regulation, values obtained reflected adequate regulation overall. However, women in the neurosurgery unit and those in the floating staff obtained the lowest scores. Statistical significance (*p*=0.023) was only observed in this area, indicating mean differences between groups.

The results indicate that health professionals exhibit a moderate fear of death, with scores ranging from 3 to 4. The lowest value was observed for the fear of one's own death, while the highest value was observed for the fear of one's own dying process and the death of others. This was particularly evident among floating staff, where the values reached high levels of fear (4.02 ± 0.99 and 4.12 ± 0.61).

**Figure 1:**
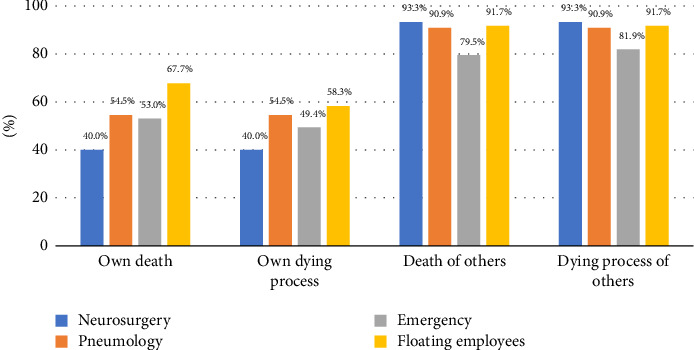
Percentage of professionals who perceive that the COVID-19 pandemic situation has increased the degree of anxiety or worry related to fear of dying in their workplace.

**Figure 2:**
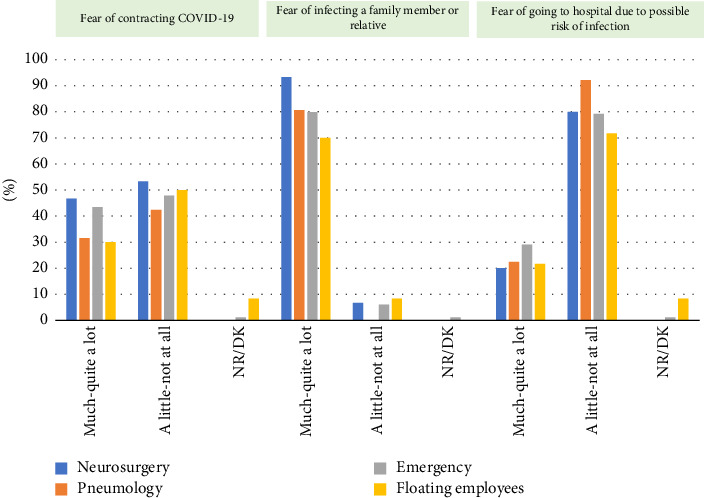
Distribution of fear levels among healthcare professionals by service areas during the COVID-19 pandemic.

As illustrated in Figure 1, the highest percentages were obtained by professionals who reported perceiving greater fear for the subscales of fear of the death of others and the process of the death of others. These differences were found to be statistically significant with respect to the other two subscales. The number of nurses in the floating staff who described perceiving the greatest fear on the latter scales was the highest.  Figure 2 displays the results obtained regarding the COVID-19 pandemic and the fear associated with various scenarios.

The relationship between the study variables and age was quantified using Pearson's coefficient.  Table 6 illustrates the association among resilience, EI, fear of death and age. Age is found to correlate with resilience, emotional regulation and the dying process of others. It can be observed that resilience is correlated with the understanding and repair of EI, as well as with the fear of the death of others. EI attention correlates with the fear of one's own death, while EI regulation correlates with the death of others.

A regression model was subsequently conducted in Table 7 to determine which variables can be considered predictors of the fear of death. This was performed using a forward multiple linear regression, the results of which demonstrate that acceptance is negatively associated with fear of death across all models, with higher levels of acceptance predicting a decrease in fear of death (*B* = −6.194 to −8.039, *p* < 0.05). In contrast, attention correlates positively with fear, with higher levels being associated with an increase in fear of death (*B* = 5.451–6.099, *p* < 0.05). Additionally, training in ethics or bioethics has been found to be negatively related to fear, with greater training in these areas being associated with lower fear (*B* = −8.205, *p* < 0.048). The final model explains 10.3% of the variability in fear of death.

## 5. Discussion

The aim of this study is to analyse the relationship among fear of death, EI and resilience among healthcare professionals during the COVID-19 pandemic. A linear regression model shows that greater acceptance and ethics training are associated with reduced fear of death.

The study data reveal a predominance of female participation, reflecting their significant presence in the professional healthcare sector [11, 18, 25, 35, 47]. The average age of the professionals in the study was 41.89 years, consistent with other studies [11]. Similarly, the average work experience was 12 years, aligning with findings from another research [35]. The study responses were primarily from nurses [36, 41, 48, 49], reflecting their predominance in the workforce during the COVID-19 pandemic [6]. The demands imposed by high hospital occupancy rates and absences related to infections or psychological issues necessitated bolstering these teams, particularly by increasing the number of floating staff [50].

### 5.1. Fear of Death

The mean levels of fear of death (3.68) are higher than those documented in research prior to the COVID-19 period [41, 51, 52]. Lázaro-Pérez et al. [53] conducted a study in Spanish hospitals during the peak of the pandemic in April 2020, reporting that 71.3% of the population studied expressed a high level of fear of the death of others. These results are also higher than those found in samples of student populations [54–57]. In contrast, even higher values (3.79) were observed in occupational therapy students who participated in an educational programme on palliative care in January and June 2022 [42]. Although these values are relatively elevated, they all fall within the range designated as medium fear.

Consistent with the FDS data, 80%–95% of the population in this study responded affirmatively to the question of whether the COVID-19 pandemic had increased their anxiety or concern regarding the fear of death. This increase was noted in the subscales of fear of dying and the dying process of others. However, as previously mentioned, the averages indicate that, while there has been an increase, it still falls within the medium fear range.

Whether perceived or measured by the FDS, the greatest fear during the pandemic and beyond is the fear of the death of others. These findings are in line with those of previous studies conducted prior to the pandemic [52, 54, 56] or during the pandemic [7, 41, 42, 53]. Notably, nurses in floating staff positions showed the highest levels of fear, on the subscales of fear of the death of others and fear of the dying process of others. This finding is consistent with two characteristics described in the scientific literature. On the one hand, the majority of the participants were female and they tended to be more emotionally affected by the process [41, 58]. Gómez-Coca et al. [18] found that individuals who experience greater fear of contracting COVID-19 tend to be more attentive to their emotional state. Other potential causes may include the fact that they are professionals who rotate through all the services and units of the hospital, are relatively young and have recently completed their studies, and have limited training and experience in this area [7, 18, 41].

As suggested by Miranda-Chavez et al. [7], the study population may experience increased fear for several reasons. One possible factor is that society prioritises discussing the death of others over one's own, as discussing one's own death remains a taboo for both professionals and Western society in general [9]. It is also important to acknowledge the unique challenges that these frontline workers face during the pandemic. They are routinely exposed to a high risk of infection and witness a significant increase in daily deaths, often in challenging circumstances [6, 18]. Furthermore, this increased fear of the death of others can be justified by the continuous contact with individuals infected with SARS-CoV-2 and the lack of personal protective equipment [59, 60].

Neurosurgery professionals, who have not been in contact with COVID-19 patients, have exhibited a significant fear of both contracting the virus and transmitting it to their families compared to their colleagues who have had direct contact with these patients. Vázquez-García et al.'s study [41] describes that emergency department staff showed lower levels of stress, depression, anxiety, fear and insomnia than professionals in other units. This contrasts with Danet's findings [6], which indicate an increase in these indicators in the ICU and pneumology service professionals.

### 5.2. EI

The values obtained for EI vary depending on the unit or service under study. A review highlights the need to examine gender differences in EI among healthcare workers [61]. Similarly, review studies indicate no significant differences between EI and professional roles [62]. The current study demonstrates significant differences in EI values by genders and service areas. The neurosurgery unit recorded the lowest EI values, particularly among female professionals. These findings align with research showing that frontline nurses during the pandemic exhibited above-average EI values, with anxiety being a prevalent negative emotion. Such negative emotions can severely impact nursing skills and care quality, leading to consequences at multiple levels [63]. Although the relationship between EI and fear of death is relatively understudied in the healthcare setting, research on medical students underscores the importance of preparing healthcare professionals for the feelings of death of others [64]. In this context, EI is essential for handling challenging situations, such as the emotions resulting from end-of-life processes, both for the family, patients and professionals themselves [20]. In line with our study's results, it was found that the attention dimension of EI was negatively related to Fear of Death Scale (FDS). This finding is consistent with the existing literature, which indicates that professionals tend to focus excessively on their emotional states, potentially leading to heightened feelings of fear of death. Our results indicate that professionals from all services demonstrated adequate scores on the EI subscales. This finding contrasts with a study conducted on nurses in the frontline of the pandemic, which reported medium–high scores and indicated anxiety as the most negative emotion [63]. In this context, EI plays a crucial role in regulating both positive and negative emotions, emphasising the need for further training in this skill. This highlights the necessity for the development of structured programmes that are specifically focussed on EI training [22, 23], as well as the incorporation of simulation-based training [24]. A recent study in China has shown that EI training not only improves EI and resilience levels in the healthcare professionals but also reduces perceived stress and contributes to a better experience for hospitalised patients. The intervention, which was carried out over 1 year, was structured in two phases. In the first phase, professionals were familiarised with the theoretical framework of EI. In the second phase, they participated in real-life case discussions in which they had to manage their emotions in the face of conflicts arising at different levels within their respective organisations [65]. Such programmes not only enhance emotional awareness and regulation but also improve the identification of emotional triggers, thereby optimising problem-solving skills.

### 5.3. Resilience

Professionals across various services, including floating staff, have showed high levels of resilience, consistent with the findings of previous studies [66]. However, floating staff exhibited a slightly lower average resilience, though without significant differences compared to non-floating staff, possibly due to their unique circumstances [67]. Resilience has been identified as a key factor in ensuring high-quality professional performance, particularly in the context of health crises such as the pandemic [68]. The research indicates that higher levels of resilience are linked to better adaptation to stressful situations [13, 69], which is crucial during pandemics in emotionally demanding environments where decisions must be made swiftly yet judiciously to uphold the quality of care and ensure the safety of both patients and healthcare professionals. In this regard, higher resilience has been found to not only enhance psychological well-being but also reduce the perception of workplace threats, thereby mitigating the impact of stress and, in this specific case, buffering the anxiety associated with COVID-19 [13]. Additionally, a positive correlation has been found among age and resilience, emotional regulation and the ability to manage others' death processes. This underscores the potential role of age and accumulated experience in strengthening these psychological attributes among healthcare professionals.

When interpreting the results of this study, it is important to consider two relevant aspects. Firstly, data collection occurred during the COVID-19 pandemic, a period when healthcare professionals' responses were shaped by the extraordinary demands of the time. This context may have influenced both the response rate and the profile of participants who completed the survey. Given the unprecedented nature of the pandemic, numerous factors may have influenced the results, some of which might not have been accounted for due to the unfamiliar context faced by both participants and researchers. These findings highlight the need for a broader approach in future studies to better capture the complexity of such situations. Secondly, the healthcare sector remains predominantly female, a trend reflected in the composition of our sample. This gender distribution limits the generalisability of the findings to male professionals. Additionally, a key limitation of this study is that the sample was drawn from a single tertiary hospital in Spain, specifically from the neurosurgery, pneumology, and emergency care departments, which played a crucial role in addressing the COVID-19 pandemic. As a result, the findings may limit the generalisability of the results to broader healthcare populations, both within Spain and internationally. Moreover, while correlations and regression models provided valuable insights, they accounted for only a small proportion of the variance. This suggests that other factors, such as personal experiences during the pandemic, family circumstances and clinical work settings, may also play a significant role. Acknowledging these limitations is crucial for understanding the scope of our research. Future studies should adopt sampling strategies that enhance representativeness (e.g., stratified sampling) and explore additional factors that could offer a more comprehensive understanding of the variables studied, particularly in complex contexts such as a pandemic, thereby improving the generalisability of the findings.

## 6. Clinical Implications

The findings of this study have the potential to result in practical applications that enhance the well-being of health professionals, especially if it is recognised that exceptional circumstances, such as a pandemic, influence the emotional responses of health workers. Firstly, professionals must strengthen skills such as EI and resilience, as these tools will help them manage both positive and negative emotions, particularly fear of death in crises such as pandemics. The high resilience scores observed in the sample may have had a modulating or protective effect on the perception of fear of death. Furthermore, the promotion of bioethics education can empower professionals to make effective end-of-life decisions. Secondly, it is imperative that healthcare management recognises the importance of supporting its staff not only technically or scientifically but also emotionally. This support must be embedded in policies that recognise that caring for professionals is equivalent to caring for patients. This approach is indicative of an investment not only in the quality of clinical care but also in prevention and the overall sustainability of the health system.

## 7. Conclusion

The findings of this study suggest that, during the challenging period of the pandemic, health professionals exhibited a high degree of resilience, as evidenced by their responses. This elevated resilience may have contributed to the observation that, despite an increase in the perception of fear of death, its levels remained in the moderate-to-high range. Additionally, the results highlight the influence of emotional awareness, training and education in bioethics on this fear. They also indicate that greater attention to emotions is associated with an increase in the perception of fear of death, while knowledge in bioethics correlates with lower values of perception of this fear.

This, in conjunction with the frequency of death and dying within the healthcare system, underscores the necessity for professionals to be adequately trained to manage these crises and end-of-life decision-making ethically and safely.

The global health crisis of the COVID-19 pandemic has emphasised the importance of equipping health professionals with technical knowledge and training in bioethics and emotional skills, such as EI and resilience. This will ensure that they are better able to cope with adversity, maintain their mental health and ensure the quality and humanity of their clinical care.

## Figures and Tables

**Table 1:** Sociodemographic and profile characteristics of respondents (*n* = 121).

Variable	Unit	Neurosurgery	Pneumology	Emergency	Floating employees	Total	
Age (mean)		39	45	43	39	42	SD 9.634
Gender		*n*	*n*	*n*	*n*	*n*	%
	Male	1	1	14		16	13.22
	Female	14	8	68	13	103	85.12
	Does not say		1	1		2	1.65
Profession							
	Doctor	2	4	8	0	14	11.57
	Nurse	12	5	51	9	77	63.64
	Nursing assistants	1	1	24	4	30	24.79
Education							
	Master's degree	2	2	18	3	25	20.66
	Doctorate	1	3	0	0	4	3.31
	In palliative care	2	2	32	6	42	34.71
	In ethics or bioethics	2	3	25	5	35	28.93
	In communicating bad news	2	2	24	2	30	24.79
Experience (average years)							
	Doctor	8	16	24		20	SD 10.69
	Nurse	14	20	14	10	14	SD 8.78
	Nursing assistants	19	15	15	13	15	SD 6.64

*Note: n*: absolute number.

Abbreviation: SD, standard deviation.

**Table 2:** Collett–Lester Fear of Death Scale (FDS-28).

Dimension	Fear measure	Population *n* = 121		
		Cutoff value	Frequency	Percentage (%)
Fear of self-death	Low	< 2	21	17.40
	Moderate	2–4	71	58.70
	High	> 4	29	24.00

Fear of self-death process	Low	< 2	2	4.10
	Moderate	2–4	69	47.90
	High	> 4	50	47.90

Fear of death others	Low	< 2	21	0.80
	Moderate	2–4	58	47.90
	High	> 4	62	51.20

Fear of process death other	Low	< 2	2	1.70
	Moderate	2–4	69	57.00
	High	> 4	50	41.30

*Note:* Frequencies and percentages are based on the cutoff points.

**Table 3:** Emotional Intelligence Scale (TMMS-24).

Dimension	Measure	Men *N* = 16			Women *N* = 103			No sex identified *N* = 2	
		Cutoff value	Frequency	% Males	Cut-off value	Frequency	% Women	Frequency	% Not ident.
Attention	Scarce	< 21	2	12.50	< 24	32	31.00		
	Adequate	22–32	11	68.80	25–35	57	55.00	2	100
	Excessive	> 33	3	18.80	> 36	14	14.00		

Clarity	Scarce	< 25	7	43.80	< 23	20	19.40	2	100
	Adequate	26–35	8	50.00	24–34	65	63.10		
	Excessive	> 36	1	6.20	> 35	18	17.50		

Repair	Scarce	< 23	2	12.50	< 23	26	25.20	1	50
	Adequate	24–35	13	81.30	24–35	61	59.20	1	50
	Excessive	> 36	1	6.20	> 35	16	15.50		

*Note:* Frequencies and percentages are based on gender and the cutoff points.

**Table 4:** Resilience scale (ER-14).

Scale	Measurement	Population *n* = 121		
		Cutoff	Frequency	Percentages (%)
ER-14 total	Very low	14–30	0	0.00
	Low	31–48	0	0.00
	Normal	43–63	4	3.30
	High	64–81	44	36.40
	Very high	82–98	73	60.30

*Note:* Frequencies and percentages are based on the cutoff points.

**Table 5:** Descriptive statistics and ANOVA results for resilience, emotional intelligence and fear of death by job title.

Job/scales	Neurosurgery	Pneumology	Emergency	Floating employees	Total	ANOVA sig.
Fear of death						
Own death	3.10 ± 1.22	2.61 ± 1.04	3.19 ± 1.02	3.23 ± 1.28	3.14 ± 1.08	0.447
Own dying process	3.90 ± 1.08	3.40 ±0 .71	3.92 ± 0.92	4.02 ± 0.99	3.89 ± 0.94	0.376
Death of others	3.92 ± 0.62	3.93 ± 0.96	3.91 ± 0.79	4.12 ± 0.61	3.94 ± 0.77	0.843
Dying process of others	3.80 ± 0.76	3.58 ± 0.76	3.79 ± 0.82	3.60 ± 0.97	3.75 ± 0.82	0.748
Total	3.68 ± 0.73	3.38 ± 0.71	3.71 ± 0.76	3.74 ± 0.77	3.68 ± 0.75	0.611

Emotional intelligence						
Attention						
Men	24 (*n* = 1)	38 (*n* = 1)	27.42 ± 5.70		27.87 ± 6.02	
Women	27.07 ± 6.41	30.50 ± 6.04	28.17 ± 5.86	30.69 ± 7.18	28.52 ± 6.14	0.191
Does not say		26 (*n* = 1)	22 (*n* = 1)		24 ± 2.82	
Understanding						
Men	23 (*n* = 1)	18 (*n* = 1)	28.21 ± 5.01		27.25 ± 5.43	
Women	27.57 ± 6.09	30.25 ± 5.92	29.04 ± 5.88	30.15 ± 5.17	29.07 ± 5.80	0.62
Does not say		25 (*n* = 1)	22 (*n* = 1)		23.5 ± 2.12	
Repair						
Men	26 (*n* = 1)	26 (*n* = 1)	27.35 ± 4.43		27.18 ± 4.15	
Women	24.00 ± 7.26	30.62 ± 4.03	28.61 ± 6.34	24.61 ± 7.14	27.64 ± 6.68	0.023
Does not say		23 (*n* = 1)	25 (*n* = 1)		24 ± 1.41	

Resilience						
Competence	66.53 ± 6.49	66.2 ± 5.84	66.72 ± 5.82	63.61 ± 7.92	66.32 ± 6.14	0.385
Acceptance	16.4 ± 2.26	16.2 ± 4.10	16.39 ± 2.94	15.15 ± 3.78	16.24 ± 3.05	0.413
Total	82.93 ± 7.63	82.4 ± 9.45	83.12 ± 7.94	78.76 ± 10.81	82.57 ± 8.37	0.598

**Table 6:** Pearson correlations: age, resilience, emotional intelligence and fear of death.

		Resilience			EI			FDS				
		Total	Competence	Acceptance	Attention	Understanding	Repair	Own death	Own dying process	Death of others	Dying process of others	Total
Age	*R*	0.277^∗∗^	0.232	0.290^∗^	−0.016	0.071	0.312^∗∗^	−0.057	0.039	−0.040	0.270^∗∗^	0.055
	Sig	0.002	0.010	0.001	0.862	0.440	0.000	0.5340.	0.674	0.663	0.003	0.548

Resilience												
Total	*R*		0.957^∗∗^	0.813^∗∗^	0.141	0.384^∗∗^	0.559^∗∗^	−0.104	−0.056	−0.215^∗^	−0.033	−0.118
	Sig		0.000	0.000	0.124	0.000	0.000	0.255	0.545	0.018	0.721	0.196
Competence	*R*			0.609^∗∗^	0.162	0.414^∗∗^	0.594^∗∗^	−0.129	−0.049	−0.232^∗^	−0.097	−0.147
	Sig			0.000	0.075	0.000	0.000	0.160	0.593	0.010	0.289	0.107
Acceptance	*R*				0.059	0.219^∗^	0.337^∗∗^	−0.027	−0.054	−0.121	0.106	−0.028
	Sig				0.523	0.016	0.000	0.768	0.559	0.187	0.248	0.758

EI												
Attention	*R*					0.451^∗∗^	0.133	0.218^∗^	0.110	0.105	0.058	0.155
	Sig					0.000	0.146	0.017	0.230	0.254	0.528	0.090
Understanding	*R*						0.423^∗∗^	−0.061	0.030	−0.040	−0.059	−0.039
	Sig						0.000	0.503	0.743	0.664	0.519	0.671
Repair	*R*							−0.156	−0.061	−0.211^∗^	−0.085	−0.152
	Sig							0.087	0.503	0.020	0.354	0.096

FDS												
Own death	*R*								0.780^∗∗^	0.487^∗∗^	0.556^∗∗^	0.877^∗∗^
	Sig								0.000	0.000	0.000	0.000
Own dying process	*R*									0.455^∗∗^	0.587^∗∗^	0.867^∗∗^
	Sig									0.000	0.000	0.000
Death of others	*R*										0.656^∗∗^	0.750^∗∗^
	Sig										0.000	0.000
Dying process of others	*R*											0.822^∗∗^
	Sig											0.000

Abbreviations: EI = emotional intelligence, FDS = fear of death scale.

**Table 7:** Regression model.

Model		Unstandardised coefficients		Standardised coefficients	*t*	Sig.	Collinearity statistics	
		*B*	Error	Beta			Tolerance	VIF
1	(Constant)	141.07	18.40		7.67	0.000		
	Acceptance	−6.19	2.98	−0.19	−2.08	0.040	1.00	1.00

2	(Constant)	129.26	18.91		6.84	0.000		
	Acceptance	−7.42	2.99	−0.22	−2.48	0.015	0.97	1.04
	Attention	5.45	2.50	0.20	2.18	0.031	0.97	1.04

3	(Constant)	133.15	18.78		7.09	0.000		
	Acceptance	−8.04	2.99	−0.24	−2.71	0.008	0.95	1.05
	Attention	6.10	2.49	0.22	2.45	0.016	0.95	1.05
	Ethics or bioethics training	−8.21	4.10	−0.18	−2.00	0.048	0.98	1.02

*Note:* Dependent variable: fear (total). For step 1: *R* = 0.035 *R*^2^ adj = 0.027; *F* 4.313 *p*=0.040. For step 2: *R* = 0.269; *R*^2^ adj = 0.072, *F* 4.600 *p*=0.012. For step 3: *R* = 0.103 *R*^2^ adj = 0.080; *F* 4.479, *p*=0.005.

## Data Availability

The data that support the findings of this study are available on request from the corresponding author. The data are not publicly available due to privacy or ethical restrictions.

## References

[1] Robles-Bello M. A., Sánchez-Teruel D., Valencia Naranjo N., Sohaib L., Sohaib L. (2022). Predictor Variables of Mental Health in the Spanish Population Confined by COVID-19. *Brain and Behavior*.

[2] Huertas C., García A. O., Cáceres E. L., Périz D. A. (2021). Del Miedo a la Resiliencia Estudio Fenomenológico Sobre el Impacto de la Pandemia Por COVID-19 en Cuidadoras de Pacientes Dependientes en Hemodiálisis. *Enfermería Nefrológica*.

[3] Kang L., Li Y., Hu S. (2020). The Mental Health of Medical Workers in Wuhan, China Dealing With the 2019 Novel Coronavirus. *The Lancet Psychiatry*.

[4] Alnazly E., Khraisat O. M., Al-Bashaireh A. M., Bryant C. L. (2021). Anxiety, Depression, Stress, Fear and Social Support During COVID-19 Pandemic Among Jordanian Healthcare Workers. *PLoS One*.

[5] Ruiz-Frutos C., Gómez-Salgado J. (2021). Efectos de la Pandemia por COVID-19 en la Salud Mental de la Población Trabajadora. *Archivos de Prevención de Riesgos Laborales*.

[6] Danet A. (2021). Psychological Impact of COVID-19 Pandemic in Western Frontline Healthcare Professionals: A Systematic Review. *Medicina Clínica*.

[7] Miranda-Chavez B., Copaja-Corzo C., Rivarola-Hidalgo M., Taype-Rondan Á. (2022). Fear of Death in Medical Students from a Peruvian University During the COVID-19 Pandemic. *Behavioral Sciences*.

[8] Silva S. M. d., Baptista P. C. P., Silva F. J. d., Almeida M. C. d. S., Soares R. A. d. Q. (2020). Resilience Factors in Nursing Workers in the Hospital Context. *Revista da Escola de Enfermagem da USP*.

[9] Conthe P. (2018). The Clinician and Their Patient at the End of Life. *Revista Clínica Española*.

[10] Lonetto R.., Templer D. I. (1986). Death Anxiety. *Series in Health Psychology and Behavioral Medicine*.

[11] Messias J. C. C., Rocha M. d O., Barbi K. B. S., Fontoura Júnior E. E., Espíndola-Fontoura E. (2022). Death and Resistance: Professionals on the Front Line Against COVID-19. *Paideia*.

[12] Sansó N., Galiana L., Oliver A., Pascual A., Sinclair S., Benito E. (2015). Palliative Care Professionals’ Inner Life: Exploring the Relationships Among Awareness, Self-Care, and Compassion Satisfaction and Fatigue, Burnout, and Coping With Death. *Journal of Pain and Symptom Management*.

[13] Bogaerts S., van Woerkom M., Erbaş Y. (2021). Associations Between Resilience, Psychological Well-Being, Work-Related Stress and COVID-19 Fear in Forensic Healthcare Workers Using a Network Analysis. *Frontiers in Psychiatry*.

[14] Mendoza Bernal I., Sánchez-Teruel D., Robles-Bello M. A., Sarhani-Robles A., Sarhani-Robles M. (2023). Predictors of Resilience in Healthcare Workers During the COVID-19 Pandemic: A Longitudinal Study Comparing the First and Second Waves. *BMC Psychology*.

[15] Salovey P., Mayer J. D. (1990). Emotional Intelligence. *Imagination, Cognition and Personality*.

[16] Lee J.-H., Sim I. O. (2021). Analysis of the Relationship Between the Psychological Well-Being, Emotional Intelligence, Willpower, and Job-Efficacy of Clinical Nurses: A Structural Model Application. *International Journal of Environmental Research and Public Health*.

[17] Chamaya C., Miguel L., Chamaya M. M. C., Saldaña S. H. G., Rengifo W. F. F., Cárdenas M. H. (2022). Tipo de Familia e Inteligencia Emocional en Enfermeros de un Hospital Público de Perú. *Enfermeria: Cuidados Humanizados*.

[18] Gómez-Coca M. M., Morocho-Sambachi S. A., Pérez-Buitrón T. G., Llanos-Román G. A. (2022). Inteligencia Emocional, Ansiedad y Miedo a COVID-19 en Voluntarios de una Organización Humanitaria. *CienciAmérica*.

[19] Jiménez-Picón N., Romero-Martín M., Ponce-Blandón J. A., Ramirez-Baena L., Palomo-Lara J. C., Gómez-Salgado J. (2021). The Relationship Between Mindfulness and Emotional Intelligence as a Protective Factor for Healthcare Professionals: Systematic Review. *International Journal of Environmental Research and Public Health*.

[20] Tavabie S., Bass S., Minton O. (2020). Emotional Intelligence in Palliative Medical Education. *British Journal of Hospital Medicine*.

[21] Theodoratou M., Papadopoulos A. (2024). Emotional Intelligence, Psychological Distress, and Conflict Resolution Among Healthcare Professionals. *European Psychiatry*.

[22] Bhattacharjee A., Ali A., Basu A. (2024). Effectiveness of Emotional Intelligence Training Programs for Healthcare Providers in Kolkata. *Educational Administration: Theory And Practice*.

[23] Maria F., Georgia F. (2024). Emotional Intelligence and Nursing Leadership. *Journal of Nursing Practice*.

[24] Logvinov Y. I., Zaitseva E. S. (2023). Development of Emotional Intelligence of Medical Workers With the Help of Simulation Training. *Virtual Technologies in Medicine*.

[25] Romero-Fernández A. J. (2024). Síndrome de Burnout y Desgaste Emocional en Personal de Enfermería’. *Revista Arbitrada Interdisciplinaria de Ciencias de La Salud. Salud y Vida*.

[26] Botello S., Mauricio C., Ganga Contreras F. A. (2021). Satisfacción Laboral y Síndrome de Burnout en Pandemia COVID-19: El Caso de Una Institución Financiera de la Zona Central de Chile. *Dilemas Contemporáneos: Educación, Política y Valores*.

[27] Cardoso N. D. C., Gomes S. D. N., Santos S. R. M. (2022). Desenvolvimento da Síndrome de Burnout em Profissionais de Enfermagem na Pandemia COVID-19. *Revista Recien-Revista Científica de Enfermagem*.

[28] Rao G. P., Koneru A., Nebhineni N., Mishra K. K. (2024). Developing Resilience and Harnessing Emotional Intelligence. *Indian Journal of Psychiatry*.

[29] Fernández-Fernández J. A., Gómez-Díaz M. (2022). Resilience and Grief Facing the Loss of a Loved One: A Systematic Review. *Journal of Psychopathology and Clinical Psychology*.

[30] Rionda I. S., Cortés-García L., Jiménez M. V. M. (2022). La Resiliencia Como Mediador Entre el Síndrome del Quemado (Burnout) y el Bienestar Subjetivo en Residentes de Hospitales Españoles. *Behavioral Psychology*.

[31] Meredith L. S., Sherbourne C. D., Gaillot S. J. (2011). Promoting Psychological Resilience in the U.S. Military. *RAND Health Quarterly*.

[32] Bergeman C. S., Blaxton J., Joiner R. (2021). Dynamic Systems, Contextual Influences, and Multiple Timescales: Emotion Regulation as a Resilience Resource. *The Gerontologist*.

[33] Quintana-Honores M. J., Isabel Vallejos A., Canova-Barrios C. J. (2023). Resiliencia en Los Profesionales de Enfermería de Una Institución Sanitaria Privada de la Ciudad Autónoma de Buenos Aires. *Enfermería Investiga*.

[34] Manzano García G., Ayala Calvo J. C. (2012). Emotional Exhaustion of Nursing Staff: Influence of Emotional Annoyance and Resilience. *International Nursing Review*.

[35] Schultz C. C., Colet C. d. F., Benetti E. R. R., Tavares J. P., Stumm E. M. F., Treviso P. (2022). Resilience and the Reduction of Occupational Stress in Nursing. *Revista Latino-Americana de Enfermagem*.

[36] Cruz-Ausejo L., Villarreal-Zegarra D., Mahony Reátegui-Rivera C. (2023). The Impact of COVID-19 Pandemic on the Quality of Life of Healthcare Workers and the Associated Factors: A Systematic Review. *Revista de Psiquiatrií y Salud Mental*.

[37] Castagnola Sánchez C. G., Cotrina-Aliaga J. C., Aguinaga-Villegas D. (2021). La Resiliencia Como Factor Fundamental en Tiempos de Covid-19. *Propósitos y Representaciones*.

[38] Viejo P., Jesús M., Dorado-Barbé A., Rodríguez-Brioso M., López-Pérez J. (2020). Resiliencia Para la Promoción de la Salud en la Crisis Covid-19 en España. *Revista de Ciencias Sociales*.

[39] Armangué A. C., Crespo J. L. (2021). Resiliencia de Profesionales Sanitarios En La Emergencia COVID-19: Ejes de Intervención. *Index de Enfermería*.

[40] Andrade A. M. G., Silva J. V. D., Baptista M. N. (2023). Psicometría Brasileña de la Escala de Miedo a la Muerte de Collett-Lester. *Revista Bioética*.

[41] Vázquez-García D., De-la-Rica-Escuín M., Germán-Bes C., Caballero-Navarro A. L. (2023). Anxiety and Fear of Death in Health Professionals in Hospital Emergency Services in Aragón. *Enfermeria Clinica*.

[42] Gutiérrez-Sánchez D., López-Leiva I., Martín-de-las-Heras S., Rubio L., Martín-Martín J. (2024). Validation of the Collett-Lester Fear of Death Scale in Occupational Therapy Students: Psychometric Testing and Implications for Palliative Care Education. *BMC Palliative Care*.

[43] Fernandez-Berrocal P., Extremera N., Ramos N. (2004). Validity and Reliability of the Spanish Modified Version of the Trait Meta-Mood Scale. *Psychological Reports*.

[44] Salovey P., Mayer J. D., Goldman S. L., Turvey C., Palfai T. P. (1995). Emotional Attention, Clarity, and Repair: Exploring Emotional Intelligence Using the Trait Meta-Mood Scale. *Emotion, Disclosure, & Health*.

[45] Sánchez-Teruel D., Robles-Bello M. A. (2018). Instrumentos de Evaluación en Inteligencia Emocional: Una Revisión Sistemática Cuantitativa. *Perspectiva Educacional*.

[46] Teruel D. S., Bello M. A. R. (2015). 14-Item Resilience Scale (RS-14): Psychometric Properties of the Spanish Version. *Revista Iberoamericana de Diagnostico y Evaluacion Psicologica*.

[47] González Z. R., Castro J. E. F., Vega G. D. L. T. (2022). Estrés Laboral en Profesionales de Enfermería de Una Unidad Quirúrgica en Tiempos de la COVID-19. *Medisan*.

[48] Kasemy Z. A., Sharif A. F., Bahgat N. M., Abdelsattar S., Abdel Latif A. A. (2023). Emotional Intelligence, Workplace Conflict and Job Burn-Out Among Critical Care Physicians: A Mediation Analysis With a Cross-Sectional Study Design in Egypt. *BMJ Open*.

[49] Simkin H., Yaccarini C., Colombano M. (2023). Depression in Healthcare Workers: Influence of Fear of Death, Spirituality, and Religion. *Revista Subjetividad y Procesos Cognitivos*.

[50] Kantorski L. P., Oliveira M. M. d., Alves P. F. (2022). Intention to Leave Nursing During the COVID-19 Pandemic. *Revista Latino-Americana de Enfermagem*.

[51] Dadfar M., Lester D. (2018). The Farsi Translation, Reliability and Validity of the Death Concern Scale. *Trends in Psychiatry and Psychotherapy*.

[52] Povedano-Jiménez M., Ropero-Padilla C., Rodriguez-Arrastia M., García-Caro M. P. (2021). Personal and Emotional Factors of Nursing Professionals Related to Coping With End-of-Life Care: A Cross-Sectional Study. *International Journal of Environmental Research and Public Health*.

[53] Lázaro-Pérez C., Martínez-López J. Á., Gómez-Galán J., López-Meneses E. (2020). Anxiety About the Risk of Death of Their Patients in Health Professionals in Spain: Analysis at the Peak of the Covid-19 Pandemic. *International Journal of Environmental Research and Public Health*.

[54] Osuna J. B., González-Serna J. M. G., Antiñolo F. M. G., Ezcurra M. Á. M. (2017). Sociodemographic Factors That Influence Death Anxiety in Medical Students. *Educación Médica*.

[55] Alonso A. I. L., Martínez M. E. F., Presa C. L., Casares A. M. V., González M. P. C. (2018). Experimental Classroom Games: A Didactic Tool in Palliative Care. *Revista da Escola de Enfermagem da USP*.

[56] Ortego-Maté C., Silió-García T., Fernández-Peña R. (2022). Sentimientos Relacionados Con la Muerte en Estudiantes de Enfermería: Un Estudio Observacional de Tres Cohortes. *Index de Enfermería*.

[57] Purimahua D. I., Manik M., Manurung E. I. (2021). Fear of Death Between Nursing Students in the Academic and Professional Programs. *Open Access Macedonian Journal of Medical Sciences*.

[58] Fernández-Martínez E., Martín-Pérez I., Liébana-Presa C., Martínez-Fernández M., López-Alonso A. I. (2021). Fear of Death and Its Relationship to Resilience in Nursing Students: A Longitudinal Study. *Nurse Education in Practice*.

[59] Mamani-Benito O., Farfán-Solís R., Tito-Betancur M., Vinelli-Arzubiaga D., Armada J., Mejía C. R. (2022). Factores Asociados a Preocupación y Miedo Durante la COVID-19 en Practicantes Preprofesionales de Salud. *Revista Cubana de Medicina Militar*.

[60] Mboua C. P., Keubo F. R. N., Fouaka S. G. N. (2021). Anxiété et Dépression Associées à la Prise en Charge de la COVID-19 Chez les Personnels de Santé au Cameroun. *L’Évolution Psychiatrique*.

[61] Nightingale S., Spiby H., Sheen K., Slade P. (2018). The Impact of Emotional Intelligence in Health Care Professionals on Caring Behaviour Towards Patients in Clinical and Long-Term Care Settings: Findings From an Integrative Review. *International Journal of Nursing Studies*.

[62] Ordoñez-Rufat P., Polit-Martínez M. V., Martínez-Estalella G., Videla-Ces S. (2021). Inteligencia Emocional de las Enfermeras de Cuidados Intensivos en un Hospital Terciario. *Enfermería Intensiva*.

[63] Sun H., Wang S., Wang W. (2021). Correlation Between Emotional Intelligence and Negative Emotions of Front‐Line Nurses During the COVID‐19 Epidemic: A Cross‐sectional Study. *Journal of Clinical Nursing*.

[64] Currey E. L., Fessler M. M., Lorenzana E., Collier K. (2022). Self-Directed Curriculum About Grief, Death, and Dying to Increase Student Emotional Intelligence. *Academic Medicine*.

[65] Mao L., Huang L. Z., Chen Q. N. (2021). Promoting Resilience and Lower Stress in Nurses and Improving Inpatient Experience Through Emotional Intelligence Training in China: A Randomized Controlled Trial. *Nurse Education Today*.

[66] Bozdağ F., Ergün N. (2021). Psychological Resilience of Healthcare Professionals During COVID-19 Pandemic. *Psychological Reports*.

[67] Rogers M., Windle A., Wu L., Taylor V., Bale C. (2024). ‘Advanced Clinical Practitioners’ Resilience and Emotional and Spiritual Well-Being During COVID-19. *Journal of Nursing Management*.

[68] Sarwar A., Naseer S., Zhong J. Y. (2020). Effects of Bullying on Job Insecurity and Deviant Behaviors in Nurses: Roles of Resilience and Support. *Journal of Nursing Management*.

[69] Awano N., Oyama N., Akiyama K. (2020). Anxiety, Depression, and Resilience of Healthcare Workers in Japan During the Coronavirus Disease 2019 Outbreak. *Internal Medicine*.

